# Inflammation in Right Ventricular Failure: Does It Matter?

**DOI:** 10.3389/fphys.2018.01056

**Published:** 2018-08-20

**Authors:** Laurence Dewachter, Céline Dewachter

**Affiliations:** ^1^Laboratory of Physiology and Pharmacology, Faculty of Medicine, Université Libre de Bruxelles, Brussels, Belgium; ^2^Department of Cardiology, Erasmus Academic Hospital, Brussels, Belgium

**Keywords:** right ventricular failure, coupling, pulmonary hypertension, inflammation, cytokines, chemokines, immune cells

## Abstract

Right ventricular (RV) failure is a common consequence of acute and chronic RV overload of pressure, such as after pulmonary embolism and pulmonary hypertension. It has been recently realized that symptomatology and survival of patients with pulmonary hypertension are essentially determined by RV function adaptation to increased afterload. Therefore, improvement of RV function and reversal of RV failure are treatment goals. Currently, the pathophysiology and the pathobiology underlying RV failure remain largely unknown. A better understanding of the pathophysiological processes involved in RV failure is needed, as there is no proven treatment for this disease at the moment. The present review aims to summarize the current understanding of the pathogenesis of RV failure, focusing on inflammation. We attempt to formally emphasize the importance of inflammation and associated representative inflammatory molecules and cells in the *primum movens* and development of RV failure in humans and in experimental models. We present inflammatory biomarkers and immune mediators involved in RV failure. We focus on inflammatory mediators and cells which seem to correlate with the deterioration of RV function and also explain how all these inflammatory mediators and cells might impact RV function adaptation to increased afterload. Finally, we also discuss the evidence on potential beneficial effects of targeted anti-inflammatory agents in the setting of acute and chronic RV failure.

## Introduction

**Figure 1:**
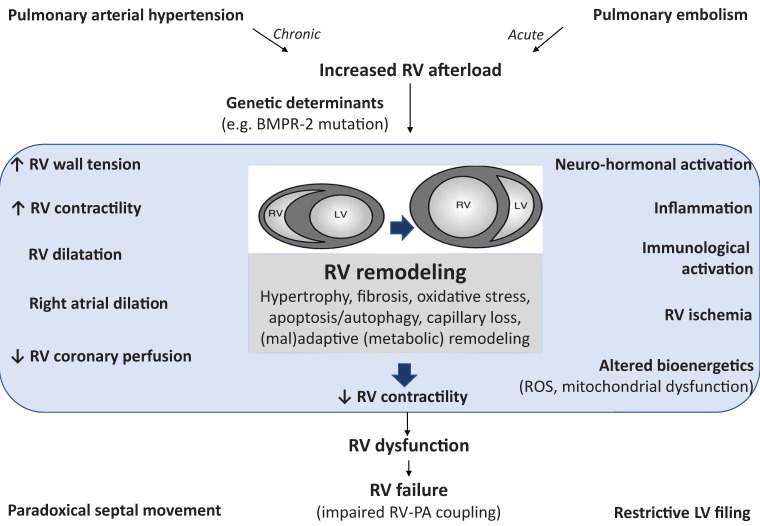
Pathophysiology of right ventricular (RV) failure implicating activation of inflammatory and immune processes. Pulmonary arterial hypertension (as well as acute pulmonary embolism) induces an increased afterload for the RV resulting in RV remodeling. The structural and functional changes during the development of RV failure can be characterized clinically, as summarized on the left hand. RV dysfunction is also probably due the activation of other pathophysiological mechanisms (summarized on the right hand) leading to multiple cellular changes, such as oxidative stress, apoptosis, inflammation, fibrosis, and metabolic remodeling. These factors also contribute to RV dysfunction and subsequent RV failure (characterized by altered coupling between the RV and the pulmonary circulation). The cellular changes are either the result of chronic RV pressure overload or the effect of circulating factors released from the sick lung circulation. RV, right ventricle; BMPR-2, bone morphogenetic protein type 2 receptor; ROS, reactive oxygen species; PA, pulmonary artery; LV, left ventricle.

Although the initial insult involves the pulmonary circulation, it has been better realized recently that symptomatology and poor clinical outcome in patients with pulmonary arterial hypertension (PAH), are essentially determined by the adaptation of right ventricular (RV) function to increased afterload (Galiè et al., 2010; Vonk-Noordegraaf et al., 2013; Vonk Noordegraaf et al., 2017; Friedberg and Redington, 2014), showing the importance to consider the coupling of the RV to the pulmonary circulation, as a sole functional unit (Naeije et al., 2014). Similarly, after pulmonary embolism, mortality and morbidity increase dramatically in patients, in presence of RV dysfunction (Kasper et al., 1997; Ribeiro et al., 1997; Kreit, 2004; Schoepf et al., 2004). We know that the RV initially adapts to an increase in afterload observed in pulmonary hypertension (PH) by an increased contractility with preserved dimensions and stroke volume

(called Anrep's homeometric adaptation). This systolic function adaptation eventually fails, resulting in increased RV dimensions (called Starling's heterometric adaptation) and decreased stroke volume (Galiè et al., 2010; Vonk-Noordegraaf et al., 2013; Vonk Noordegraaf et al., 2017; Friedberg and Redington, 2014). Cellular and molecular mechanisms underlying the development of RV dysfunction (from adaptive to maladaptive processes) remains insufficiently understood (Figure 1). Moreover, specific pharmacologic therapy that can reverse RV failure is not yet available and the effects on RV function of available PAH therapies remain largely elusive.

In recent years, activation of inflammatory processes has been identified as one of the major pathogenic components of pulmonary vascular remodeling, contributing to the development of various forms of pulmonary PH (Humbert et al., 2004; Rabinovitch, 2012; Voelkel et al., 2016). Additionally, circulating levels of inflammatory mediators, such as interleukin (IL)-6, IL-1β, tumor necrosis factor (TNF)-α, and monocyte chemoattractant protein (MCP)-1, have been shown to be elevated in PAH and correlated to the severity of the disease (Humbert et al., 1995; Dolenc et al., 2014). However, the role of inflammation in the transition from RV adaptation to RV failure is still poorly understood.

**Figure 2:**
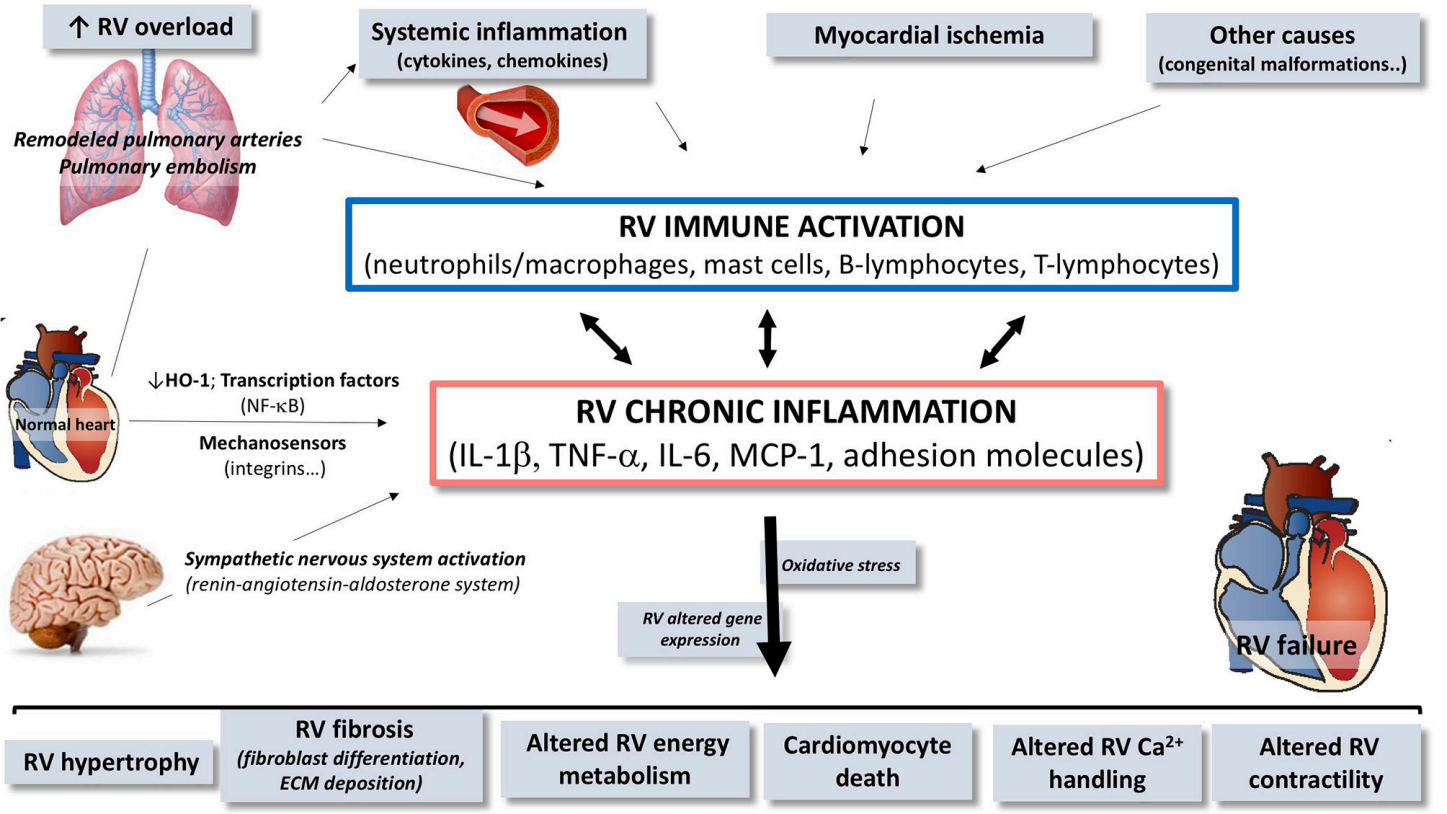
Schematic overview of the contribution of immune and inflammatory mediators and cells in the pathogenesis of right ventricular (RV) dysfunction/failure. In presence of pulmonary arterial hypertension or pulmonary embolism, circulating inflammatory mediators originating from the pulmonary vasculature may trigger or contribute to local activation of inflammatory processes in the RV. Inflammation in the RV is characterized by activation of immune processes, implying expansion and activation of different cell types and by up-regulation of cytokines, chemokines, and cell adhesion molecules contributing to chronic inflammatory status. Through this upregulation of immune and inflammatory processes, mediators contribute to the development of RV failure. RV, right ventricle; HO-1, heme oxygenase-1; NF-κB, nuclear factor kappa B; IL, interleukin; TNF, tumor necrosis factor; MCP, monocyte chemoattractant protein; ECM, extracellular matrix.

Described in many different cardiovascular diseases (other than PAH) (Mann, 2002; Frangogiannis, 2014), myocardial inflammation has progressively emerged as a pathophysiologic process contributing to cardiac hypertrophy, fibrosis and dysfunction in heart failure (Frieler and Mortensen, 2015; Mann, 2015). In patients with idiopathic PAH (Overbeek et al., 2008; Condliffe et al., 2009) or selected forms of congenital heart diseases, such as Eisenmenger syndrome (Kuhn et al., 2003), RV failure is less prevalent and occurs later compared to patients with PAH associated to inflammatory diseases such as systemic sclerosis (Kawut et al., 2003; Kuhn et al., 2003; Overbeek et al., 2008; Condliffe et al., 2009). In these patients, RV inflammatory infiltrates were denser than in patients with idiopathic PAH, while interstitial fibrosis was similarly present in all the RV (Overbeek et al., 2010). This strongly suggests that RV failure is predominant in patients with PH presenting an inflammatory background. However, the implication of inflammation to RV dysfunction is suspected in all forms of PH, including both chronic and acute increase in RV afterload (Iwadate et al., 2003; Begieneman et al., 2008; Overbeek et al., 2010). In experimental models of RV failure, myocardial inflammation has also been described, with increased infiltration of inflammatory cells and expression of various cytokines and chemokines (Campian et al., 2010; Rondelet et al., 2012). We also know that, in the heart, inflammatory processes are inextricably linked to cell death, oxidative stress, altered cell metabolism and extracellular matrix remodeling, which all have been incriminated in the pathogenesis of RV failure (Figures 1, 2; Bogaard et al., 2009a).

In the present review article, we propose an overview of the multiple players involved in the complex inflammatory response to acute or chronic increased afterload and its contribution to subsequent (mal)adaptive remodeling of the RV leading to RV dysfunction, regarding what's already known in the left ventricle (LV) and in heart failure in general.

## Inflammation and RV failure

Inflammation is an essential biological stimulation-response system provided by the immune system to ensure the survival after noxious stimuli, such as infection or tissue injury. Inflammatory responses may, therefore, be considered as a classic homeostatic system, functioning to maintain normal organ function. In the heart, activation of inflammatory processes induced by sterile stressors is largely similar to that observed during infection, including the release of vasoactive peptides, the expression of adhesion molecules [e.g., vascular cell adhesion molecule (VCAM)-1, intercellular adhesion molecule (ICAM)-1] in cardiac cells (e.g., cardiomyocytes, fibroblasts, endothelial cells) that promote myocardial recruitment of inflammatory cells (e.g., neutrophils, macrophages, lymphocytes), the release of inflammatory cytokines and chemokines, and the activation of T cell-mediated adaptive immune responses (Chen and Nuñez, 2010; Frieler and Mortensen, 2015; Mann, 2015; Prabhu and Frangogiannis, 2016). This initial response to harmful stimuli represent *acute inflammation*, which probably evolves as an adaptive response to restore tissue homeostasis and function. However, when this harmful inflammatory trigger persists, it can cause dramatic tissue damage eventually leading to cardiac loss of function (Libby, 2007). This prolonged dysregulated and maladaptive response of the body is *chronic inflammation*, which involves myocardial inflammation, tissue destruction and attempts to repair tissue damages, leading to altered myocardial function.

**Figure 3:**
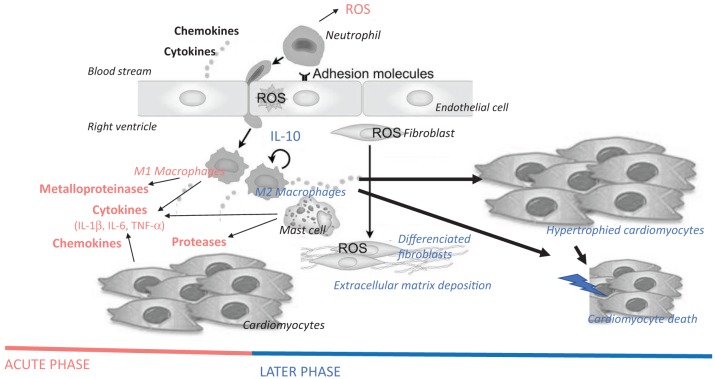
Hypothetical schematic cellular and molecular insights of immune cells and inflammatory mediators on the pathogenesis of right ventricular failure, more precisely on cardiomyocyte hypertrophy and death, as well as on fibrosis. Immune and inflammatory cells are myocardial resident cells or are coming from the blood stream, adhere to endothelial cells and invade the myocardium. Cytokines (e.g., IL-1β, IL-6, and TNF-α), chemokines (e.g., MCP-1), and enzymes (e.g., metalloproteinases and proteases, such as chymase) released by these cells promote cardiomyocyte hypertrophy, fibroblast differentiation/activation, deposition of extracellular matrix and cardiomyocyte apoptosis. Acute-phase inflammatory and immune mediators are represented in red, while late-phase mediators after myocardial injury are represent in blue. ROS, reactive oxygen species; IL, interleukin; TNF, tumor necrosis factor.

Accumulating evidence suggests that all cardiac cell types could participate to this inflammatory response by their own and therefore playing a central vicious role in the maintenance of these maladaptive processes associated to chronic inflammation (Van Linthout et al., 2014), leading to heart failure. RV activation of inflammatory processes is associated with and contributes to RV adverse remodeling and dysfunction (Campian et al., 2010; Rondelet et al., 2012; Dewachter et al., 2015). In RV failure, an elevated expression of cytokines and chemokines modulate various intracellular signaling pathways in cardiac cells, leading to cardiomyocyte hypertrophy and death, mitochondrial dysfunction, endoplasmic reticulum stress, and cardiac fibrosis characterized by fibroblast proliferation and differentiation and collagen deposition (Figure 3; Frieler and Mortensen, 2015; Mann, 2015; Prabhu and Frangogiannis, 2016). In addition, these inflammatory mediators also alter myocardial metabolic processes and cardiomyocyte contractile properties.

## Mediators and effectors of inflammation—cytokines and chemokines

Cytokines play a crucial role in inflammatory response to acute myocardial injury, mediating the recruitment of inflammatory and immune cells into the injured area (Seta et al., 1996) and exerting direct detrimental effects on the heart (Seta et al., 1996). Released directly by the heart itself (Shimano et al., 2012), cytokines can also be produced by cardiomyocytes (Kapadia et al., 1995), cardiac endothelial cells (Liu et al., 2014) and fibroblasts, resident macrophages (Pinto et al., 2016) and infiltrated inflammatory cells, as well as by extra-cardiac tissues (e.g., adipose tissue). In PAH patients, increased levels of circulating pro-inflammatory cytokines (e.g., IL-1β, TNF-α) have been correlated to the severity of the disease (Humbert et al., 1995; Dolenc et al., 2014), which also reinforces the vicious circle between inflammation and RV failure.

### IL-1 signaling pathway

In persistent RV failure induced by acute pulmonary artery banding, IL-1β was overexpressed in the RV, mainly in the vessels and myocardial infiltrating cells, rather than in cardiac cells themselves (Dewachter et al., 2015). This was associated with decreased expression of IL-33, a cardio-protective cytokine of the IL-1 family, and of ST2, a soluble decoy receptor regulating negatively the IL-1/IL-33 signaling (Dewachter et al., 2015). Overexpression of IL-1α and−1β was also reported in experimental RV failure on chronic systemic-to-pulmonary shunting in pigs (Rondelet et al., 2012), while IL-33 expression did not change (Belhaj et al., 2013). Pro-inflammatory cytokines (such as IL-1β and TNF-α) act as acute-phase mediators after tissue injury. In the heart, they mediate negative inotropic effects and promote myocardial hypertrophy and cell death (Bujak and Frangogiannis, 2009). Mechanistically, IL-1β-induced negative inotropic effects were mediated by inducible nitric oxide (NO)-synthase activation and peroxynitrite production, which can interfere with the excitation-contraction coupling, leading to a rapid and reversible contractile dysfunction (Finkel et al., 1992; Van Tassell et al., 2013). In addition, it was demonstrated that IL-1β (as well as IL-6) were able to reduce the expression of sarco/endoplasmic reticulum calcium (Ca^2+^)-ATPase in cardiomyocytes (Thaik et al., 1995), suggesting abnormalities of Ca^2+^ handling underlying these negative inotropic effects. IL-1β promotes cardiac hypertrophy through insulin-like growth factor-1 release from cardiac fibroblasts, via a paracrine mechanism involving signal transducer and activator of transcription3 (STAT3) activation (Honsho et al., 2009). In cardiomyocytes, IL-1β-induced apoptosis was reported to be mediated by the activation of inducible NO-synthase and the upregulation of Bcl2 homologous antagonist/killer (Bak) and B-cell lymphoma-extra-large (Bcl-XL) (Ing et al., 1999). In addition, IL-1β directly induced cardiomyocyte growth in a NO-independent manner (Thaik et al., 1995). On the other hand, IL-1β also contributed to fibrotic processes, stimulating the release of matrix metalloproteinases in cardiac fibroblasts, through the inhibition of the endoglin signaling and the activation of the bone morphogenetic protein (BMP) pathway (Saxena et al., 2013). IL-1β, has also been shown to impair cardiac energy metabolism, increasing myocardial oxygen demand adding to the detrimental effects of myocardial contractile performance in a context of oxygen supply limitation (Hofmann et al., 2007). Therefore, upregulation of IL-1β could contribute to the pathogenesis of RV failure by decreasing RV contractility, inducing cardiomyocyte death and altered supply in metabolic energy.

### TNF-α signaling pathway

RV failure induced by transient pulmonary artery banding was associated with a RV increase in TNF-α expression, whereas circulating serum levels of TNF-α remained undetectable after pulmonary artery banding (Dewachter et al., 2010), suggesting local early expression of this pro-inflammatory cytokine in this experimental model. A local increased expression of TNF-α was also observed in experimental brain death-induced RV dysfunction (Belhaj et al., 2016) and tightly correlated to the prediction to develop RV failure early after heart transplantation (Birks et al., 2000). Expression of TNF-α was increased in the failing RV after 6-month systemic-to-pulmonary shunting in pigs, together with increased circulating serum TNF-α levels (Rondelet et al., 2012). Early activation of inflammatory processes, characterized by an increase in TNF-α and myeloperoxidase expression, was also observed in RV dysfunction in rats developing severe PH induced by monocrotaline injection (Campian et al., 2010). In patients with advanced heart failure, myocardial expression of TNF-α was abundantly increased (Torre-Amione et al., 1996), probably contributing to maladaptive mechanisms implicated in the development of cardiac hypertrophy and dysfunction (Frieler and Mortensen, 2015; Mann, 2015; Prabhu and Frangogiannis, 2016). In these patients, high circulating TNF-α levels were tightly correlated to myocardial fibrosis, inflammation, as well as ventricular dilatation and mortality (Kubota et al., 2000b). This is consistent with experimental data showing that TNF-α contributes to substantial cardiac remodeling. In cardiomyocytes, TNF-α induced the activation of apoptosis (Chandrasekar et al., 2004), through the activation of nuclear factor-κB (NF-κB) signaling (Glezeva and Baugh, 2014). TNF-α induced cardiomyocyte hypertrophy, through mechanisms dependent on the interaction between cell integrin and the extracellular matrix (Yokoyama et al., 1997) and the activation of AKT/NF-κB and JNK pathways (Higuchi et al., 2006). In cardiac fibroblasts, TNF-α induced proliferation and collagen production through the suppression of miR-29 (Venkatachalam et al., 2009). Experimentally, TNF-α has been shown to contribute to systolic and diastolic dysfunction (Kubota et al., 2000a; Dibbs et al., 2003) and increased arrhythmogenesis (Lee et al., 2007). In mice, cardiomyocyte-specific overexpression of TNF-α induced dilated cardiomyopathy characterized by ventricular hypertrophy and dilatation, myocardial infiltration of inflammatory cells, fibrosis and cardiomyocyte apoptosis, together with reduced ejection fraction (Kubota et al., 1997). In contrast, TNF-α-deficient mice subjected to pressure overload were protected against cardiac hypertrophy, fibrosis and dysfunction (Sun et al., 2007). Interestingly, TNF-α (as well as IL-1β) downregulated the expression of Ca^2+^-regulating genes, including sarcoplasmic reticulum Ca^2+^ ATPase (Wu et al., 2011) and Ca^2+^-release channels (Thaik et al., 1995), responsible for direct negative inotropic effects (Yokoyama et al., 1993; Duncan et al., 2007). This shows the contribution of inflammation in Ca^2+^ imbalance and cardiac remodeling, leading to a vicious circle observed in heart failure (Tschöpe and Lam, 2012). Moreover, TNF-α also altered the activity of β-adrenergic receptors, leading to the uncoupling of these receptors from the adenylyl cyclase (Gulick et al., 1989; Chung et al., 1990). In RV failure, TNF-α could, therefore, contribute to altered RV contractility and remodeling, through a Ca^2+^ imbalance responsible for ventriculo-arterial uncoupling. Moreover, it has been suggested that estrogen could alter inflammatory cell-induced synthesis of TNF-α, preventing the induction of cardiac fibroblasts that leads to adverse remodeling of the extracellular matrix (McLarty et al., 2013). This could, at least partly, explain why estrogen contributes to RV function improvement in different experimental models of PH (Frump et al., 2015; Liu et al., 2017).

### IL-6 signaling pathway

In experimental RV failure, increased expression of IL-6 in the RV was inversely correlated to RV adaptation to increased afterload [assessed by the ratio between the pulmonary arterial elastance (Ea) and the end-systolic elastance (Ees)], while RV expressions of binding- (IL-6R) and signal transducing (gp130)-subunits of the IL-6 receptor remained unchanged (Dewachter et al., 2015). This is well-known that IL-1 and TNF-α are able to induce IL-6 release in different cell types (Zhang et al., 1990), which suggests that IL-6 has direct negative inotropic effects (Yu et al., 2005), but can also potentate these of IL-1 and TNF-α (Maass et al., 2002). Therefore, increased RV expression in IL-6, IL-1β, and TNF-α could contribute to the amplified activation of inflammatory processes in the setting of RV failure. Circulating IL-6 levels were increased in RV failure on acutely increased afterload (Dewachter et al., 2015), which also probably contributes to the perpetuation of the inflammatory state and to the acceleration of the progression of global heart failure (as already described in the setting of LV failure). High circulating levels of CRP and IL-6 were independently associated to increased RV mass and volume (Harhay et al., 2013). High circulating levels of IL-6 have been reported in patients with severe PAH (Humbert et al., 1995), predicting negatively their survival and outcome (Soon et al., 2010). In contrast, RV expression of IL-6 did not change in RV failure induced by 6-month systemic-to-pulmonary shunting in pigs (Rondelet et al., 2012). Therefore, IL-6 could play a role in the transition from acute to chronic inflammation in RV failure. Moreover, IL-6 has also been incriminated in the pathogenesis of cardiac hypertrophy and dysfunction (Frieler and Mortensen, 2015; Mann, 2015; Prabhu and Frangogiannis, 2016), through the activation of the Ca^2+^/calmodulin-dependent protein kinase II and STAT3 pathways (Kunisada et al., 1996). Experimentally, infusion of IL-6 in rats was able to induce cardiac hypertrophy, inflammation, fibrosis and diastolic dysfunction (Meléndez et al., 2010), whereas IL-6 genetic deletion ameliorated angiotensin II- (Coles et al., 2007; Ma et al., 2012) and norepinephrine-induced cardiac hypertrophy and fibrosis (Meier et al., 2009). Downregulation of IL-6 expression reduced inflammation and reversed altered glucose metabolism induced by high fat diet in mice, through the inhibition of suppressor of cytokine signaling-3 signaling and the upregulation of insulin receptor substrate-1 signaling (Ko et al., 2009). This suggests that chronic inflammation may contribute to cardiac dysfunction through metabolic perturbations that can impair cardiac energetic production in response to metabolic stress. Overexpression of the signal transducer gp130 was shown to be sufficient to induce cardiomyocyte hypertrophy and to mediate IL-6 effects (Ancey et al., 2003), through the activation of STAT3 signaling (Kunisada et al., 1998) and the GRB2-associated-binding protein 1-Src homology 2 domain-containing phosphatase2 interaction (Nakaoka et al., 2003). Therefore, IL-6 could also be implicated in the development of RV remodeling in RV failure, but its precise role should be confirmed for the RV.

### IL-10 signaling pathway

In RV failure on acutely increased afterload, the pro-inflammatory status (described above) is reinforced by the downregulation of anti-inflammatory cytokine IL-10 (Dewachter et al., 2015), leading to increased pro-inflammatory ratio of IL-6/IL-10 both in the RV and in the serum. Macrophages are the major source of IL-10, a cytokine that mediates its anti-inflammatory effects, through the inhibition of the synthesis of various inflammatory molecules such as interferon–γ, IL-1, IL-6, and TNF-α (Anker and von Haehling, 2004). Therefore, IL-10 is usually called a neutralizer of inflammation and a tissue protective cytokine. However, expression of IL-10 did not change in the failing RV in an experimental model of systemic-to pulmonary shunting in pigs (Rondelet et al., 2012), suggesting a role mainly in the acute phase of inflammation. The role of IL-10 in heart failure is not well established. In experimental myocardial infarction, myocardial IL-10 expression was decreased (Kaur et al., 2006). Circulating IL-10 levels were diminished in patients with heart failure (Stumpf et al., 2003), while it has been reported that elevated levels of IL-10 and TNF-α was associated with an increased risk of mortality (Amir et al., 2010). These data are conflicting. Nevertheless, IL-10 is considered as a cardio-protective cytokine and increased levels of IL-10 in heart failure may be seen as a compensatory mechanism to counter deleterious effects of cytokines, such as TNF-α or IL-6.

### MCP-1 (endothelium-derived CC chemokine ligand 2; CCL-2) signaling pathway

Expressions of MCP-1 and its receptor CCR2 were increased in the failing RV in two experimental models of RV failure on acute increase in afterload (Watts et al., 2006; Dewachter et al., 2015). Increased expression of MCP-1 (as well as other chemokines) has been described in the pressure-overloaded RV following pulmonary artery banding and linked to altered expression of small leucine-rich proteoglycans by cardiac cells (e.g., fibroblasts), probably contributing to matrix remodeling (Waehre et al., 1985) and inflammation regulation (Iozzo and Schaefer, 2010; Moreth et al., 2010). Circulating levels of MCP-1 were increased in patients with PAH (Sanchez et al., 2007) and with heart failure (Kohno et al., 2008). However, the precise mechanistic role played by MCP-1 in heart failure remains elusive. We know that chemokines are, at least, able to promote myocardial infiltration and activation of leukocytes in the failing heart. Via its receptor CCR2, MCP-1 induced cardiomyocyte apoptosis, therefore contributing to ventricular dysfunction (Zhou et al., 2006). Interestingly, myocardial expression of MCP-1 increased during the early phases of myocardial infarction (Maekawa et al., 2004; Hayasaki et al., 2006) and inhibition of MCP-1 prevents ventricular remodeling after myocardial infarct (Hayashidani et al., 2003). Moreover, targeted deletion of MCP-1 in mice was shown to improve survival, attenuate LV dilatation and reduce contractile dysfunction after coronary occlusion (Hayashidani et al., 2003). In contrast, myocardial overexpression of MCP-1 was associated with altered contractile function associated with myocardial infiltration of leukocytes, mainly macrophages (Kolattukudy et al., 1998). Moreover, MCP-1 also induced the release of pro-inflammatory cytokines (Wrigley et al., 2011), such as IL-1β and IL-6, participating to a “cytokine cascade” leading to the amplification of inflammatory processes in RV failure.

## Cellular regulators of inflammation

### Heme oxygenase (HO)-1

Expression of HO-1 was decreased in the failing RV following acute (Dewachter et al., 2015) and chronic increase in afterload (Belhaj et al., 2013), with a tight correlation between RV expression of HO-1 and RV-pulmonary artery coupling (assessed by the Ees/Ea ratio) (Belhaj et al., 2013; Dewachter et al., 2015), suggesting a functional role of HO-1 in maintaining RV systolic function. This stress-inducible enzyme plays crucial roles in the control of inflammation and cytoprotective processes (Otterbein et al., 1999). Indeed, HO-1 catalyzes heme degradation into carbon monoxide, biliverdin and iron (Tenhunen et al., 1968). Through the biological activities of its metabolite products, activation of HO-1 contributes to cell defense, through reduced oxidative stress and inhibition of the activation of inflammatory and apoptotic processes. Moreover, carbon monoxide is an effective vasodilator which is also able to inhibit platelet aggregation, reduce leucocyte adhesion, cellular apoptosis, and pro-inflammatory cytokine production. Therefore, a decrease in HO-1 expression may lead to an increase in pro-inflammatory cytokine expression (Constantin et al., 2012) in the failing RV. Moreover, there were inverse relations between HO-1 expression and RV neutrophil and macrophage infiltration, as well as with RV pro-apoptotic Bax/Bcl-2 ratio (Dewachter et al., 2015), which strongly suggests a potential mechanistic link between downregulated HO-1 expression and inflammation and apoptosis in RV failure. In chronic hypoxia-exposed mice, administration of mesenchymal cells overexpressing HO-1 was associated with reduced RV hypertrophy, limited infarcted zones and decreased RV systolic pressure to normal values (Liang et al., 2011). In contrast, in the same experimental PH model, downregulation of HO-1 was shown to induce severe RV dilatation and dysfunction, together with cardiac inflammation, fibrosis, and apoptosis (Yet et al., 1999). However, expression of HO-1 was respectively increased and decreased in the RV of experimental models of RV pressure overload (Katayose et al., 1993) and RV failure (Bogaard et al., 2009b). This suggests variable HO-1 expression depending on the stress-induced cardiomyocyte damage and the progression of RV failure. In RV failure, we could speculate that downregulated HO-1 expression could impair its physiological implication in the control of inflammation activation in RV failure. However further studies are necessary to confirm that.

### NF-κB

In an inflammatory experimental model of PH, cardiac specific inhibition of the major inflammatory transcription factor NF-κB, prevented RV hypertrophy and remodeling, despite the presence of PH, mainly through the restored expression of BMP signaling members, and the reduced inflammatory phenotype (including reduced expression of IL-6 and cell adhesion molecules) (Kumar et al., 2012). Activation of NF-κB signaling also plays regulates cardiomyocyte hypertrophy, promoting cardiomyocyte growth and expression of fetal sarcomeric genes, whereas its inhibition reduces cardiac growth *in vivo* (Kawano et al., 2005; Zelarayan et al., 2009; Liu et al., 2012). Mechanistically, the cross-talk between NF-κB and nuclear factor of activated T-cells (NFAT) seems to be critical to promote cardiomyocyte growth (Liu et al., 2012). In addition, cardiac expression of peroxisome proliferator-activated receptor gamma coactivator-1α, a master regulator of mitochondrial function (Shah et al., 2016), has been shown to be inhibited by chronic inflammatory activation, through a NF-κB-dependent mechanism, suggesting that chronic activation also impairs mitochondrial metabolic regulation. However, the precise role of NF-κB in the progression of RV failure remains unknown. Activation of NF-κB has been associated to the development of heart failure in both humans and experimental models. Myocardial levels of NF-κB were increased in patients with advanced heart failure (Frantz et al., 2003). In contrast, in patients with advanced heart failure with LV assist device, the number of NF-κB immune-positive myocardial cells decreased (Grabellus et al., 2002), suggesting that activation of NF-κB signaling seems to involve a complex cellular response to heart failure.

### Cellular mechanosensing

In heart failure, wall stress increases, exposing cardiac cells to increasing biomechanical strain. Mechanosensitive adhesion proteins, including integrins, and cadherins, transduce these mechanical signals, and can stimulate inflammation (Schroer and Merryman, 2015). It has been described, in stretched cardiomyocytes and in hemodynamic-overloaded myocardium, increased secretion of TNF-α and IL-6, together with increased expression of atrial natriuretic peptide (Yoshida et al., 2014). Upon mechanical stretch, cardiac fibroblasts, rather than cardiomyocytes themselves, can be activated, secreting more chemokines and inflammatory cytokines (such as IL-1β), but also extracellular matrix components (Lindner et al., 2014). This contributes to recruit further monocytes by allowing transendothelial migration into cardiac tissue (Lindner et al., 2014). Mechanical strain induce in macrophages the activation of inflammatory processes, leading to increased expression of TNF-α, IL-6, and metalloproteinases acting on the extracellular matrix (Pugin et al., 1998) and increased expression of scavenger receptors (Sakamoto et al., 2001). Moreover, macrophages submitted to mechanical strain are more prone to entry the cell cycle (Sager et al., 2016), suggesting increased wall tension observed in right heart failure could participate to local macrophage proliferation. Strong similarities suggest that all these phenomena could be of importance in RV failure, but still remain unexplored.

## Inflammatory and immune cells

In heart failure, the inflammatory/immune component includes infiltrated neutrophils/monocytes, macrophages, dendritic cells, and lymphocytes, but also cardiac resident cells such as cardiomyocytes, fibroblasts, and endothelial cells (Figure 2). All these cells are responsible for local cardiac expression and release of inflammatory mediators (Figure 3).

After injury, circulating immune cells, which come from lymphoid organs (spleen and bone marrow), are directed to sites of injury, adhere (or come close) to endothelial cells, invade the myocardium and release a variety of inflammatory molecules (e.g., cytokines and chemokines), acting locally, and promoting chemotaxis of other inflammatory cells. These released cytokines induce the activation of inflammatory processes, mediating multiple interactions between circulating and cardiac cells (Kim et al., 2014). These complex communications result in cardiac remodeling through matrix deposition (mainly collagen) and remodeling, cardiomyocyte apoptosis and differentiation. In addition, we know that inflammatory cells activate cardiac fibroblasts leading to adverse deposition of extracellular matrix, which contributes to the pathobiology of heart failure.

### Innate immune cells

#### Dendritic cells

Acting as sentinels, immature dendritic cells patrol the blood and peripheral tissue to detect foreign and pathogenic antigens, as well as tissue damage and inflammation. This leads to antigen phagocytosis by dendritic cells, which then expresses the maturation marker CD83 and class I and II major histocompatibility complexes. Mature antigen-presenting dendritic cells migrate to secondary lymphoid tissue, where they present antigens to naïve helper and cytotoxic T-cells and prime them (Banchereau et al., 2000). According to their hematopoietic origin, dendritic cells can be divided into myeloid and plasmacytoid dendritic cells, inducing a Th1 and Th2-biased immune response respectively. Furthermore, specialized cardiac dendritic cells have been found in the human heart (Zhang et al., 1993; Yokoyama et al., 2000), and further characterized as a subtype of dendritic cells expressing human leukocyte antigen-DR (but not S100, CD1a, CD21, CD23, and CD35) (Yokoyama et al., 2000). This different surface marker profile compared to ordinary dendritic cells has led to the hypothesis that dendritic cells could change their phenotype depending on the local environment. In various cardiovascular diseases (as well as in hypoxic conditions), dendritic cells play a central role in mediating immunological effects (Yilmaz et al., 2009; Kretzschmar et al., 2015; Rohm et al., 2016). In end-stage heart failure, elevated numbers of dendritic cells have been identified, with a marked increase in myeloid dendritic cells and a concomitant decrease in plasmacytoid dendritic cells (Athanassopoulos et al., 2004, 2009). This suggests a systemic Th1 polarization in these patients. In contrast, lower circulating myeloid and plasmacytoid dendritic cell counts have been described in decompensated heart failure (Sugi et al., 2011). In idiopathic PAH patients, decreased percentage of monocyte-derived dendritic cells has been observed in the peripheral blood, suggesting a Th1 reaction in these patients (Wang et al., 2009). However, the presence and the potential role of dendritic cells have not been considered yet in RV failure.

#### Mast cells

Mast cells are granulocytes that develop in the bone marrow and migrate, with the blood stream, to different tissue, where they differentiate and mature. Upon inflammatory stimuli, cardiac mast cells degranulate, releasing a broad spectrum of mediators, including histamine, leukotrienes, growth factors, vasoactive substances, proteases, and cytokines (i.e., IL-1, TNF-α). The secretion of mast cell content is responsible for local inflammation. During the last decade, the possible role of cardiac mast cells has emerged in the pathogenesis of various cardiovascular diseases (Levick et al., 2011). Indeed, increased number of mast cells has been documented in hypertensive and failing LV (Batlle et al., 2006) and described as playing an important role in LV fibrosis, hypertrophy and failure (Stewart et al., 2003; Levick et al., 2008; Zhang et al., 2011). Therapy with mast cell stabilizer reduced fibrosis and preserved LV wall mass in experimental fulminant myocarditis in rats (Mina et al., 2013). Prolonged pressure overload on the RV induced by pulmonary artery banding was associated with increased mast cell density in the RV (Olivetti et al., 1989). This probably results from proliferation and maturation of resident immature cardiac mast cells (Forman et al., 2006; Li et al., 2012), as well as from recruitment of mast cell progenitors followed by further maturation and differentiation in the RV (Frangogiannis et al., 1998; Ngkelo et al., 2016). Despite the presence of RV hypertrophy, mast cell density was not affected in the RV of 3-month-old rats born at high altitude (Rakusan et al., 1990). Mast cells also contribute to cardiac hypertrophy and fibrosis by synthesizing and secreting pro-hypertrophic and pro-fibrotic cytokines (e.g., TNF-α and IL-6) and growth factors [e.g., transforming growth factor (TGF)-β and basic fibroblast growth factor] (Gordon and Galli, 1990; Gordon et al., 1990; Shiota et al., 2003; Sun et al., 2007; Meléndez et al., 2010). In addition, mast cells can also promote tissue fibrosis, stimulating proliferation, maturation and synthesis of collagen in cardiac fibroblasts (Liao et al., 2010). Upon their degranulation, masts cells release very high levels of proteases (e.g., chymases and tryptases) which can activate the proliferation and the synthesis of matrix protein in fibroblasts (Cairns and Walls, 1997; Akers et al., 2000). Inhibition of these proteases was shown to prevent the development of cardiac fibrosis and improve LV dysfunction in experimental models of LV disease (Matsumoto et al., 2003; Kanemitsu et al., 2006). Therefore, inhibition of mast cell proteases might be an original strategy to manage cardiac function. In the hypertrophied RV induced by pressure overload, the expressions of mast cell proteases (i.e., chymases-2,-4,-5,-6 and exopeptidase CPA3) were upregulated (Luitel et al., 2017). Moreover, a mast cell stabilizing compound was tested in chronic hypoxia exposed rats, showing significant reduced RV hypertrophy and lung mast cell hyperplasia (Kay et al., 1981). This should be further explored in RV failure.

#### Neutrophils/monocytes and macrophages

Upon tissue damage, monocytes massively leave the blood stream to differentiate into macrophages in tissues. There, they patrol to eliminate dead cells or pathogens, using phagocytosis and destroying foreign bodies by enzymatic digestion. Macrophages also reside in many healthy tissues, with a substantial tissue-specific heterogeneity among each macrophage population. In the heart, 6–8% of non-cardiomyocytes are cardiac resident macrophages (Pinto et al., 2016), with a dynamic balance between classically-activated macrophages (M1-like cells) and alternatively-activated macrophages (M2-like cells) depending on the activation stimulus. M1 macrophages are known to display a cytotoxic and pro-inflammatory phenotype characterized by strong pathogen and debris clearance and pro-inflammatory cytokine (i.e., IL-6, TNF-α, IL-1β, IL-12, and IL-23) secretion. In contrast, M2 macrophages suppress immune and inflammatory responses (through the release of anti-inflammatory IL-10 and TGF-β), and participate in tissue remodeling and scar formation (Frantz and Nahrendorf, 2014). Deriving from local cardiac progenitors, M2 macrophages have been shown to be the major steady-state cardiac macrophage population, even if their specific functions remain largely unknown. However, these cells may have typical tissue resident macrophage roles, including guarding against infection/insult, but also probably regulating cardiac metabolism, contraction, extracellular matrix deposition, and survival of cardiomyocytes (Frantz and Nahrendorf, 2014). In experimental models of myocardial infarction (Swirski et al., 2009) and chronic heart failure (Ismahil et al., 2014), activation and migration of monocytes to the heart have been described and tightly linked to the activity of angiotensin II (Leuschner et al., 2010). In the early inflammatory steps after myocardial injury, M1 macrophages are predominantly present, while during the later remodeling phase, M2 macrophages are mostly present. M1 macrophages probably act to clear debris, dead cardiac cells and neutrophils in order to allow tissue regeneration. This initial phase is followed by a proliferation phase during which M2 macrophages participate to myocardial mechanical stability through the regulation of angiogenesis and myofibroblast activity (Nahrendorf et al., 2007). The presence of sequential biphasic M1/M2 macrophage response seems to be crucial for wound healing and for a stable myocardial scar after cardiac injury (van Amerongen et al., 2007; Frantz et al., 2013). However, overabundant pro-inflammatory macrophages are also harmful. Indeed, massive recruitment of macrophages to the heart in response to cardiac injury has a prominent role in the development of myocardial remodeling, hypertrophy and fibrosis (Zhang et al., 2011; Frieler and Mortensen, 2015; Mann, 2015; Prabhu and Frangogiannis, 2016). During the progression of the pulmonary hypertensive disease, the role, dynamics and composition of M1/M2 macrophage populations in the RV are currently largely undefined. Most data are descriptive and come from *post-mortem* histological analysis of RV obtained after fatal pulmonary thromboembolism. They showed RV inflammatory infiltrate predominantly comprised of macrophages, T cells (Begieneman et al., 2008; Orde et al., 2011), neutrophils and macrophages (Iwadate et al., 2003). In experimental pulmonary embolism in rats, early and acute RV damage was associated with infiltration of mononuclear cells with characteristics of M1 phenotype. In the later phase, RV contractile function was reduced together with RV infiltration of mononuclear cells with M2 phenotype and collagen deposition beginning scar formation. This strongly suggests that neutrophil response corresponds to the early acute phase of inflammatory events, while macrophages are present during the proliferative phase and extracellular matrix deposition, changing from M1 to M2 phenotype (Watts et al., 2008). In an experimental model of persistent RV failure on acute increase in afterload, RV extravascular macrophage number was increased and tightly correlated to the coupling of the RV to the pulmonary circulation (assessed by the Ees/Ea ratio) (Dewachter et al., 2015), suggesting a potential mechanistic link between RV macrophage infiltration and RV dysfunction. Ischemia, which can be present in RV failure, induces the recruitment of macrophages (through MCP-1 release) (Kai et al., 2005). This could, at least partly, explain why RV dysfunction after pulmonary embolism was associated with increased expression of MCP-1 and C-C motif chemokine ligand 3 (CCL3 or MIP-1α), as well as RV infiltration with neutrophil and monocyte/macrophage (Watts et al., 2006). In the hypertrophied RV, increased number of activated macrophages contributes to the release of a variety of pro-inflammatory cytokines (e.g., MCP1 and metalloproteinases), that contribute to the pathogenesis of RV failure. To date, cardiac macrophages have never been therapeutically targeted. Crucial to maintain the steady sate and defending against infection, specific population of macrophages should be targeted to avoid collateral damage. During inflammatory processes, monocyte recruitment, which is tightly regulated by interaction between CCL2 and CCR2, could be reduced using silencing of the chemokine CCR2 with nanoparticles, as already experimentally tested in experimental myocardial infarction (Majmudar et al., 2013). It should be interesting to evaluate this further in RV failure.

### Adaptive immune cells

#### B-lymphocytes

B-lymphocytes are able to differentiate into antibody-producing plasma B-cells, which play crucial roles in cell-mediated immune regulation through antigen presentation, cytokine release, differentiation of T-effector cells, and collaboration with antigen-presenting dendritic cells. In PAH patients, a distinct gene expression profile of their peripheral blood B-lymphocytes has been identified (Ulrich et al., 2008b), suggesting activation of B-cells in these patients. Moreover, antibodies directed against pulmonary endothelial cells and fibroblasts have been found in PAH, suggesting a role of B-cells in the pathogenesis of PAH (Tamby et al., 2005, 2006). However, the role played by these cells in the pathogenesis of RV failure remains unknown.

#### T-lymphocytes

T-lymphocytes play a central role in cell-mediated immunity and include different types regarding their activity. T helper cells type 1 (Th1) are mainly pro-inflammatory and induce macrophage activation, while T helper cells type 2 (Th2) are predominantly anti-inflammatory through the release of multiple anti-inflammatory cytokines, such as IL-4,-10,-13. Treg cells control the balance between Th1 and Th2 responses, and are implicated in the control of autoimmunity. Tregs not only control other T-cells but also regulate monocytes, macrophages, dendritic cells, natural killer cells and B-cells. In heart failure, the presence of circulating CD4+ T-cells (expressing inflammatory cytokines) tightly correlates with altered LV function (Satoh et al., 2006; Fukunaga et al., 2007), and probably contributes to the transition from cardiac adaptation to heart failure. B- and T-lymphocyte-deficient mice submitted to chronic pressure overload had preserved systolic function, reduced myocardial fibrosis and macrophage infiltration (Laroumanie et al., 2014). In contrast, increased number of CD4+ T-cells have been reported early after coronary occlusion in mice (Hofmann et al., 2012), probably contributing to wound healing just after ischemic injury. Moreover, CD4+ T-cell deficient mice presented impaired wound healing with extracellular matrix disorganization in the ischemic zone (Hofmann et al., 2012). This strongly suggests protective adaptive immune responses early after myocardial insult, which seem to be detrimental at latter stages. Altered Treg function has been identified in patients with PAH (Tamosiuniene et al., 2011; Huertas et al., 2012) and in patients with PAH associated to HIV, systemic sclerosis, systemic lupus erythematosus, Hashimoto's thyroiditis, Sjögren's syndrome, and the anti-phospholipid syndrome (Speich et al., 1991; Covas et al., 1992; Mandl et al., 2004; Bonelli et al., 2009; Radstake et al., 2009). Moreover, higher numbers of circulating Treg cells have been shown in PAH patients, suggesting altered immune control by CD4+ T-lymphocytes (Ulrich et al., 2008a). However, the proportion of these cells in the hypertrophied/failing RV remains to be investigated in PAH, as well as their role in the pathogenesis of RV failure.

### Resident cardiac cells

#### Endothelial cells

In cardiac endothelial cells, pro-inflammatory cytokines induce the expression of adhesion molecules (Tamaru et al., 1998), promote subsequent endothelial binding of immune cells and platelets (Zakrzewicz et al., 1997) and transendothelial migration (Woodfin et al., 2009). In an experimental model of RV failure induced by transient pulmonary artery banding, RV expression of VCAM-1 increased, while expression of ICAM-1 did not change (Dewachter et al., 2015). This was associated to increased RV expression of cytokines and chemokines, together with RV infiltration of neutrophils and macrophages, indicating an early immune response. RV dysfunction associated to brain death was also associated to increased expression of ICAM-1,-2, and VCAM-1 in the RV (Stoica et al., 2005; Belhaj et al., 2016), suggesting endothelial activation, which persists in the post-operative period, even in the absence of acute rejection. On the other hand, we also know that cytokines induce the apoptosis of cardiac endothelial cells and increase endothelial generation of reactive oxygen species (ROS), which induce the production of plasminogen activator inhibition-1 and of collagen by cardiac endothelial cells (Chandrasekar et al., 2004).

## Inflammation as potential therapeutic target in right heart failure

Even if inflammatory processes are activated in RV failure, the precise role of inflammation is yet to be deciphered in this morbid condition. Indeed, it remains unknown if inflammation in the RV could be a key transition step from RV adaptation to failure or if RV activation of inflammatory processes could be a mere bystander and a normal consequence to the primary processes involved in the pathogenesis of RV failure. In addition, whether a myocardial inflammatory process is exclusively maladaptive or whether it may be protective in allowing the heart to properly respond to metabolic stress remains elusive in RV failure. It was previously demonstrated that after acute myocardial injury, an acute inflammatory phase is important to remove damaged tissue and to induce the repair mechanisms that lead to scar formation (Frieler and Mortensen, 2015; Mann, 2015; Prabhu and Frangogiannis, 2016). Suppression of this acute inflammatory phase was proven to be detrimental and to impair post-infarction remodeling (van Amerongen et al., 2007; Frantz et al., 2013). Therefore, further studies should be focused on elucidating the various phases implicated in the pathogenesis of RV inflammation, and trying to dissect the mediators and cellular components that are important in each of these phases. On the other hand, we also know that clinical trials evaluating specific anti-inflammatory treatment in heart failure, despite a strong pathobiological background also present in this condition, were, so far, quite disappointing (Glezeva and Baugh, 2014; Hartman et al., 2018), which makes one wonder if the appropriate signals are being targeted.

While the activation of inflammatory processes has been obviously identified in RV failure secondary to PH, targeting some of them may prove ineffective or offer an unacceptable risk-to-benefit ratio. For example, while TNF-α appears to be clearly implicated in the pathogenesis of RV failure, as well as of PAH, antagonizing this cytokine was not convincing with mixed pre-clinical results (Henriques-Coelho et al., 2008; Wang et al., 2013) and it is known to predispose patients to severe infectious complications such as tuberculosis. In PAH patients, clinical studies targeting such inflammatory mediator and evaluating the effects on RV function have not yet been performed. Moreover, we know that currently approved drugs used to treat PAH patients, which did not primarily target inflammation, have been shown to present some anti-inflammatory effects in pre-clinical and clinical studies (Stasch et al., 2011; Stitham et al., 2011; Fontoura et al., 2014; Dewachter et al., 2015). However, there is a paucity of data evaluating the effects of these drugs on the RV *per se*. Therefore, this is really difficult to discriminate the potential beneficial RV effects of these drugs vs. those observed in the pulmonary circulation. In an experimental model of acute RV failure on pulmonary embolism, selective anti-inflammatory therapy targeted at neutrophil chemoatractants present beneficial effects on RV function, reducing RV inflammation and damage (Zagorski et al., 2007).

Right heart failure is associated with an increase in sympathetic nervous system tone and an activation of the renin-angiotensin-aldosterone system, both resulting in fluid retention and vascular and myocardial remodeling. In patients with severe PAH, we also know that neuro-humoral activation is associated with a decreased survival (Ciarka et al., 2010; de Man et al., 2013a,b). The activation of the adrenergic nervous system and the regulation of the production of cytokines are tightly linked. Indeed, the activation of β2-receptors reduced TNF-α expression, while its increased anti-inflammatory IL-10 production (Ng and Toews, 2016). Conversely, α1,2- adrenergic stimulation increased expression of TNF-α and reduced in IL-10 (Spengler et al., 1990). We could therefore speculate that blocking the renin-angiotensin system may induce anti- inflammatory effects and may therefore result in reduced structural and functional alterations in the RV.

## Conclusions and future perspectives

In conclusion, the role of inflammation in the development of RV failure appears to be significant. This is true for both RV failure on acute and chronic increase in afterload. However, more effort is needed to understand the mechanisms promoting this pathologic process and how to modulate it in order to develop new therapeutic interventions aiming at the reduction of RV failure and mortality in PAH patients. Moreover, it seems that inflammation and RV failure are strongly interconnected and mutually reinforce each other. Therefore, the inflammatory processes should be counteracted at early stages to stop the vicious circle existing between inflammation and heart failure. As illustrated in Figure 1, it may be that RV failure is not a uniform disease but a clinical syndrome, where different pathways play different important roles. More frequent RV failure in patients with PAH due to chronic inflammatory disorders compared to patients with idiopathic PAH, might be a potential RV affection secondary to systemic inflammation. But if so, the question arises why the LV becomes not altered under these conditions.

Based on the results of experimental and translational studies presented here, we could speculate that a better understanding of the pathogenesis of RV failure will open the door for new therapeutic targets. Probably, different stimuli at various stages in the development of RV failure trigger inflammation, ROS generation, mitochondrial metabolism alteration and induction of cell death. Therefore, it is important to point out key regulators of these signaling pathways. Inflammation and ROS generation may not necessarily be harmful in RV failure, and may even play a protective role depending on the trigger (acute or chronic increase in afterload) and the context. However, uncontrolled or excessive exposure of tissues to intense inflammatory signals may be detrimental. The relevance in the control of inflammation warrants further investigation in RV failure to evaluate if the use of anti-inflammatory therapy to improve RV function might be useful.

## Author contributions

All authors listed have made a substantial, direct and intellectual contribution to the work, and approved it for publication.

### Conflict of interest statement

The authors declare that the research was conducted in the absence of any commercial or financial relationships that could be construed as a potential conflict of interest.
